# Resolving Fine Cardiac Structures in Rats with High-Resolution Diffusion Tensor Imaging

**DOI:** 10.1038/srep30573

**Published:** 2016-07-28

**Authors:** Irvin Teh, Darryl McClymont, Rebecca A. B. Burton, Mahon L. Maguire, Hannah J. Whittington, Craig A. Lygate, Peter Kohl, Jürgen E. Schneider

**Affiliations:** 1 Division of Cardiovascular Medicine, Radcliffe Department of Medicine, University of Oxford, Oxford, OX3 7BN, United Kingdom; 2 Department of Physiology, Anatomy and Genetics, University of Oxford, Oxford, OX1 3PT, United Kingdom; 3 National Heart and Lung Institute, Imperial College London, London, SW3 6NP, United Kingdom; 4 Institute for Experimental Cardiovascular Medicine, University Heart Centre Freiburg · Bad Krozingen, Medical School of the University of Freiburg, Freiburg, 79110, Germany

## Abstract

Cardiac architecture is fundamental to cardiac function and can be assessed non-invasively with diffusion tensor imaging (DTI). Here, we aimed to overcome technical challenges in *ex vivo* DTI in order to extract fine anatomical details and to provide novel insights in the 3D structure of the heart. An integrated set of methods was implemented in *ex vivo* rat hearts, including dynamic receiver gain adjustment, gradient system scaling calibration, prospective adjustment of diffusion gradients, and interleaving of diffusion-weighted and non-diffusion-weighted scans. Together, these methods enhanced SNR and spatial resolution, minimised orientation bias in diffusion-weighting, and reduced temperature variation, enabling detection of tissue structures such as cell alignment in atria, valves and vessels at an unprecedented level of detail. Improved confidence in eigenvector reproducibility enabled tracking of myolaminar structures as a basis for segmentation of functional groups of cardiomyocytes. *Ex vivo* DTI facilitates acquisition of high quality structural data that complements readily available *in vivo* cardiac functional and anatomical MRI. The improvements presented here will facilitate next generation virtual models integrating micro-structural and electro-mechanical properties of the heart.

Traditional histo-anatomical methods have laid the foundation for understanding heart structure^1^, as condensed in a number of modern cardiac models^2^. However, debate continues regarding the discretisation of myocytes into anatomical and functional groups^2^,^3^, as well as their distribution into myolaminar structures^2^,^4^,^5^,^6^,^7^, both of which are thought to play important roles in the contraction of the heart^8^. Due to excellent resolution and cell-type specificity, histological methods form a ‘gold standard’ for tissue characterisation. At the same time, they suffer from tissue distortion during sample preparation, usually limited spatial coverage, and non-trivial challenges during reconstruction of two-dimensional (2D) data stacks into three-dimensional (3D) volumes^9^.

Diffusion tensor imaging (DTI)^10^, in contrast, enables high-resolution 3D structural mapping of the whole heart, including assessment of locally prevailing cell orientations (referred to as ‘fibres’) and myolaminar tissue structures (‘sheetlets’)^7^,^11^. In DTI, the diffusion of water can be represented by a 2^nd^ order tensor. The orientations and lengths of the three orthogonal major axes of this tensor are described by its primary, secondary and tertiary eigenvectors (**v**_**1**_, **v**_**2**_, **v**_**3**_) and eigenvalues (λ_1_, λ_2_, λ_3_) respectively. The former have been shown to correspond to the fibre long-axis, sheetlet and sheetlet-normal directions respectively in myocardial laminae^7^,^11^, as validated by histology^11^,^12^,^13^.

The availability of high quality, non-distorted *ex vivo* DTI data is invaluable to the field of computational modelling of the heart. Recent developments in computational models of heart function utilised *ex vivo* DTI data for prediction of properties such as myocardial stiffness^14^, ventricular pressures, and cardiac geometry, kinematics and function^15^,^16^,^17^. Such models could assist pre-surgical planning and predict outcomes of invasive interventions. They could in conjunction with *in vivo* dynamic data, inform on patient-specific tissue mechanical properties with important implications for diagnosis and treatment^15^.

However, obtaining robust DTI measurements at high spatial resolution remains a challenge, even *ex vivo*. Current limitations of DTI include poor signal-to-noise ratio (SNR), long acquisition times^18^, sensitivity to gradient calibration^19^ and temperature variations^20^, and difficulty distinguishing **v**_**2**_ from **v**_**3**_in the presence of noise^21^. To overcome these obstacles, we implemented a set of refinements to increase SNR and spatial resolution, minimise orientation bias in diffusion-weighting, and reduce temperature variation in DTI. The improvement in data quality enabled identification of fine anatomical features not previously described with DTI, and novel segmentation of cardiomyocyte populations.

## Results

The technical refinements are first assessed in terms of image SNR, and fractional anisotropy (FA) and 95% cone-of-uncertainty (COU) of the DTI data. Eigenvector tracking and relevant angle maps are subsequently quantified.

### Dynamic receiver gain

**Figure 1:**
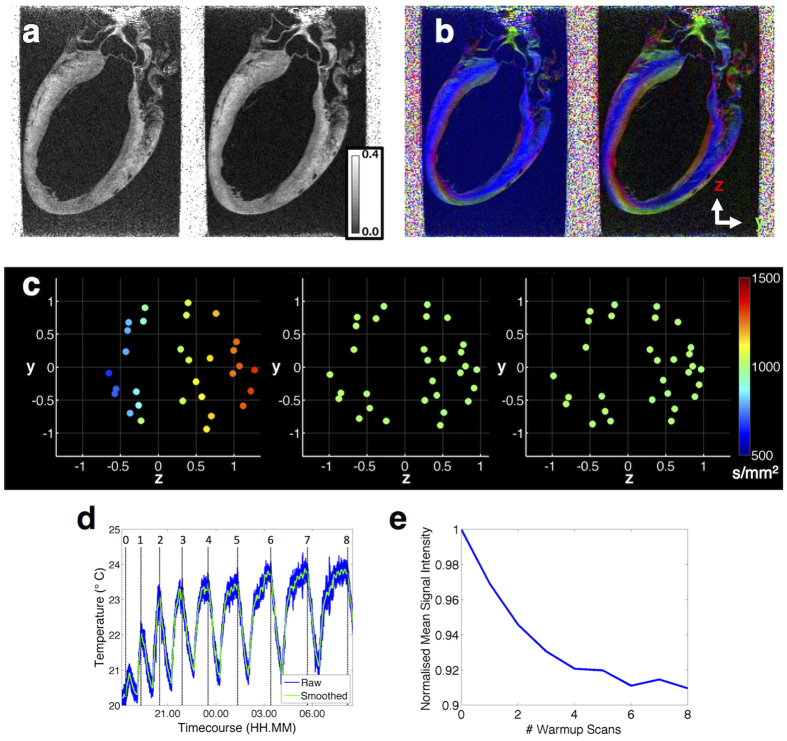
Technical refinements in DTI. **(a)** FA maps, in a mid-sagittal plane, show improved SNR when acquired with dynamic (right) over static (left) receiver gains. **(b) v**_**1**_ maps reconstructed from offset (left) and calibrated (right) data illustrate how poor gradient calibration leads to bias in **v**_**1**_. **(c)** Projection of the 3D diffusion-weighting scheme in the y-z plane without adjustment (left), with reversed diffusion gradient correction (middle) and with prospective diffusion gradient adjustment (right). Each point represents the diffusion-weighting in a single direction along the x, y and z-axes, normalised by the nominal b-value of 1,000 s/mm^2^. These plots are colour-coded by the effective b-value, including contributions from diffusion gradients, imaging gradients and cross-terms. The unadjusted scheme shows a bias towards higher/lower b-values along the +ve/−ve direction with respect to the refocusing crusher gradients (+z), as reflected by the colour variation across the plot. The proposed prospective diffusion gradient adjustment ameliorates this bias, as per the reversed diffusion gradient method, but without the associated doubling in acquisition time. **(d)** Timecourse of temperature measurements over multiple warm-up and cooldown cycles. Warm-up scans consisting of 0 to 8 DW scans preceded each b = 0 image acquisition. Start times of b = 0 acquisitions denoted by numbers 0 to 8. Scans were paused for 30 min after each b = 0 acquisition to allow samples to cool. **(e)** Mean signal intensity variation in the myocardium in the whole heart in non-diffusion-weighted (b = 0) datasets after 0 to 8 warm-up diffusion-weighted scans. The rising temperature led to a drop in b = 0 image intensity, reaching a steady state after about 6 warm-up scans.

The higher receiver gain in the diffusion-weighted (DW) scans increased the myocardial signal intensity about four-fold, improving estimates of DTI parameters (Fig. 1a). SNR and 95% COU averaged across the myocardium were as follows: SNR_static gain_ (non-DW/DW) = 47/13, SNR_dynamic gain_ (non-DW/DW) = 47/24, 95% COU_static gain_ (**v**_**1**_/**v**_**2**_/**v**_**3**_) = 10.4°/26.9°/25.2° and 95% COU_dynamic gain_ (**v**_**1**_/**v**_**2**_/**v**_**3**_) = 6.0°/15.8°/15.0°. The smaller 95% COU values in the latter reflect the improved precision in eigenvector estimates.

### Gradient system calibration

The results in the offset scan, simulating poor calibration, showed that **v**_**1**_ estimates were biased towards the x-direction of the scanner, and that the FA was artefactually enhanced in the buffer within the left ventricle cavity (Fig. 1b): FA_buffer, offset_ = 0.13 ± 0.02, FA_buffer, calibrated_ = 0.04 ± 0.02, 95% COU_myocardium, offset_ (**v**_**1**_/**v**_**2**_/**v**_**3**_) = 8.2°/15.7°/13.5° and 95% COU_myocardium, calibrated_ (**v**_**1**_/**v**_**2**_/**v**_**3**_) = 6.0°/15.8°/15.0°. The mean ADC in the global myocardium was reduced from 1.3 ± 0.3 mm^2^/s to 1.1 ± 0.2 mm^2^/s in the offset scan (mean ± standard deviation across all voxels in the myocardium). In contrast, FA increased from 0.19 ± 0.07 to 0.22 ± 0.07 in the offset scan. There was also increased heterogeneity, with higher artefactual FA occurring where **v**_**1**_ of the diffusion tensor corresponded to the direction with the most positive relative gradient bias, i.e. x-direction. HA is more tightly distributed in the offset scan at 0.7° ± 41.1° compared to −0.2° ± 49.0° suggesting a less linear transmural progression. SE and SA are particularly sensitive metrics and marked regional variation can be seen between the offset and calibrated scans (Supplementary Figure S1).

### Prospective diffusion gradient adjustment

In the assessment of the prospective adjustment of diffusion gradients, it was found that the resulting b_effective_ ranged from 666–1, 320 s/mm^2^ in the uncorrected scan, and from 999–1, 001 s/mm^2^ in both the reversed diffusion gradient and prospective adjustment methods (Fig. 1c). Measurements of precision in the whole myocardium were 95% COU_uncorrected_ (**v**_**1**_/**v**_**2**_/**v**_**3**_) = 9.0°/23.4°/21.9°, 95% COU_reversed diffusion gradient_ (**v**_**1**_/**v**_**2**_/**v**_**3**_) = 7.6°/18.9°/17.5° and 95% COU_prospective adjustment_ (**v**_**1**_/**v**_**2**_/**v**_**3**_) = 7.3°/19.0°/17.8°.

### Interleaving DW and non-DW scans

The investigation of temperature effects showed that the sample temperature at the start of each non-DW scan ranged from an ambient 20.2 °C without warm-up scans to 23.8 °C following a series of warm-up scans (Fig. 1d). There was cyclical heating during the warm-up DW scans and cooldown during the 30 min imaging pause, reaching a steady state peak of 23.8 °C after 6 warm-up DW scans. There was an inverse relationship between sample temperature and mean myocardial signal intensity in the non-DW images, where the latter was elevated by up to 10% when acquired without warm-up scans (Fig. 1e). As a result of the applied warm-up scans, the 1 standard deviation variation in mean global signal intensity across the 8 non-DW repetitions in the DTI scans ranged from 0.8% to 1.2% in each of the five hearts.

### Eigenvector tracking

**Figure 2:**
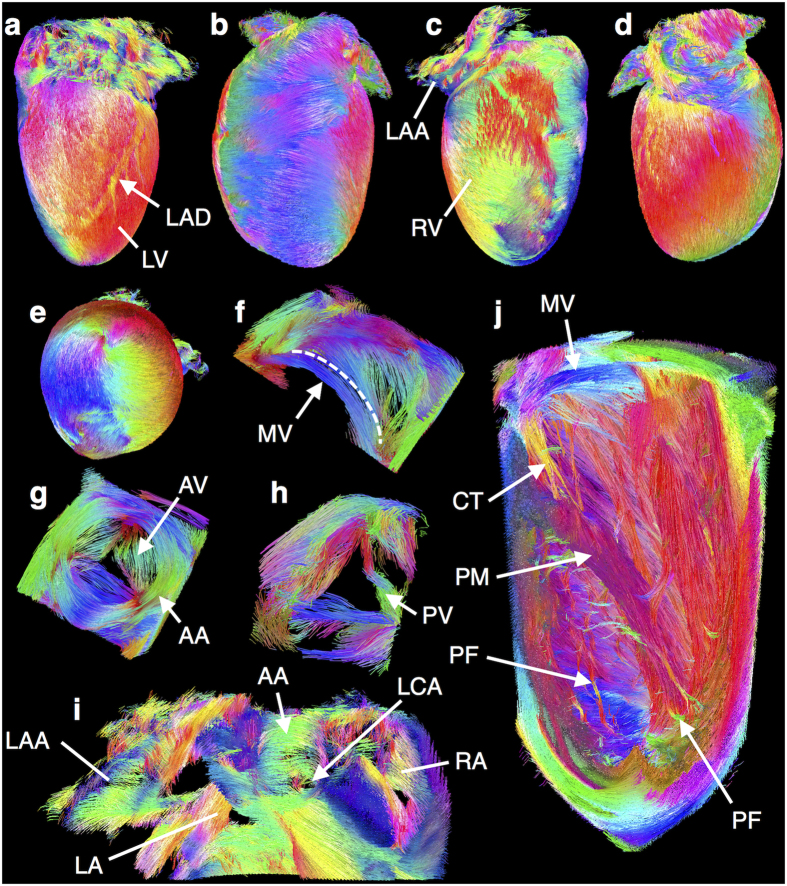
Eigenvector tracking in the whole heart. Tracks were reconstructed based on **v**_**1**_ and colour-coded by orientation: apico-basal (red), anterior-posterior (green) and lateral-septal (blue). Tracks in the heart from **(a)** lateral, **(b)** anterior, **(c)** septal, **(d)** posterior and **(e)** apical views. Left helical tracks of the LV subepicardium and circumferential tracks of the RV myocardium are seen. Gaps in subepicardial tracks correspond to the left anterior descending artery (LAD). Tracks can be seen to spiral towards the apex. **(f)** Tracks in the leaflets of the mitral valve (MV) are primarily oriented parallel to the boundary between leaflets (dashed line). **(g)** Tracks in the vessel wall of the aorta (AA) are aligned primarily in a circumferential direction, while in the cusps of the aortic valve (AV) they are oriented in an equilateral configuration before curving basally into their insertions into the aortic wall. **(h)** The pulmonary valve (PV) comprises three leaflets, with tracks organised in an equilateral configuration. **(i)** Tracks in the left and right atria (LA and RA), and left atrial appendage (LAA) viewed from the septal wall. The left coronary artery (LCA) is seen extending from the aorta and tracks in its vessel walls are aligned circumferentially. **(j)** Tracks in a 13 × 5 × 5 mm section in the LV reveal chordae tendinae (CT), oriented longitudinally between their connections to the mitral valve and papillary muscle (PM). Networks of Purkinje fibres (PF) can be seen towards the apex.

Tracks in the whole heart reconstructed from **v**_**1**_ are illustrated in Fig. 2a–e. ‘Tracks’ are defined as streamlines connecting contiguous and similarly oriented eigenvectors. Tracks based on **v**_**1**_reflect cell orientations in the underlying biological structure. The left-handed helical orientation of cells in the left ventricle (LV) lateral and posterior subepicardial walls is evident. Gaps in tracks on the subepicardium are caused by large coronary vessels. Along the subepicardium of the LV anterior wall and the right ventricle (RV), tracks are almost circumferential, with a population of longitudinal tracks in the RV basal subepicardium. Tracks are seen to spiral clockwise towards the apex. A selection of small features, not previously identified in rodent hearts by DTI, are highlighted (Fig. 2f–h). Tracks in the mitral valve are oriented roughly parallel to the free edges of anterior and posterior leaflets (Fig. 2f). Circumferentially oriented tracks are seen in the aortic wall, while in cusps of the aortic valve they align, parallel to the free edge of each cusp, curving basally towards their insertion into the aortic wall (Fig. 2g). The three leaflets of the pulmonary valve are visible, with tracks positioned in a similar fashion to the aortic valve (Fig. 2h). Tracks in the left and right atria, and left atrial appendage are identified more clearly in cross-sectional view (Fig. 2i). Tracks in the left coronary artery, and other major vessels, are oriented circumferentially as in the aortic wall. Tracks in the papillary muscles are aligned with the long-axis of the papillary muscles (Fig. 2j). Tracks in the chordae tendinae, comprised of well-aligned collagen, elastin and endothelial cells, run along the connections between mitral valve and papillary muscle attachment points, demonstrating that diffusion anisotropy can be observed in the absence of cardiomyocytes. Tracks extending from the apical myocardium along the LV endocardium appear to reflect free-running Purkinje fibres. Identification of putative Purkinje fibres was corroborated with 33 μm isotropic anatomical MRI data, acquired in the same session (Supplementary Figure S2).

**Figure 3:**
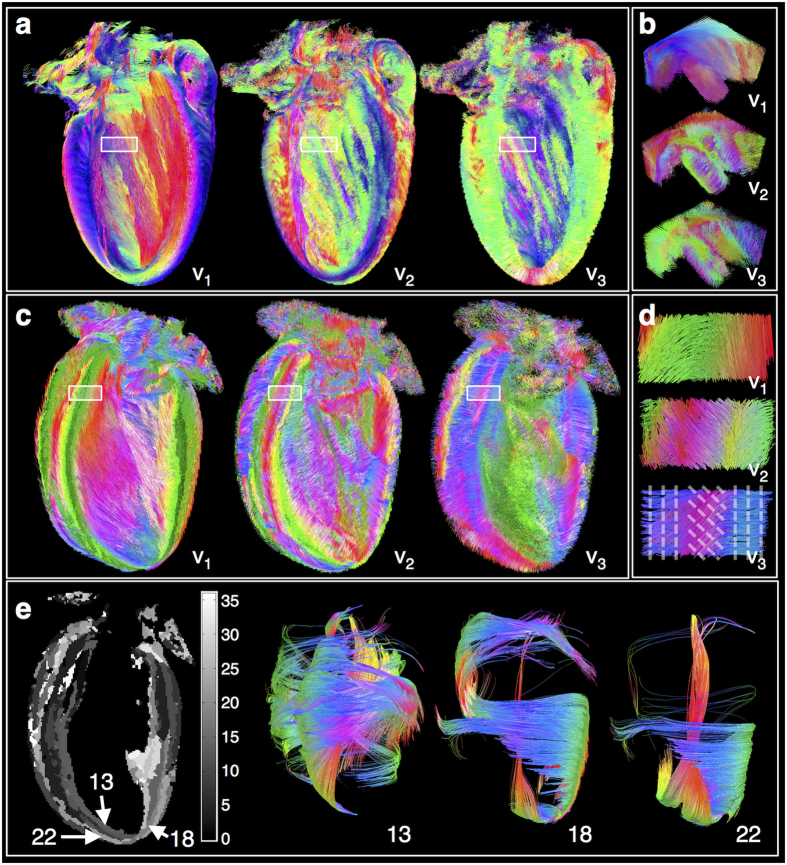
Tracking and segmentation of myocardial cell and sheetlet structures. Tracks were reconstructed based on **v**_**1**_, **v**_**2**_ and **v**_**3**_ and colour-coded by orientation: apico-basal (red), anterior-posterior (green) and lateral-septal (blue). **(a)** Digitally bisected heart viewed from the LV septal wall. **(b)** Magnified isometric view of tracks in a 4 × 3.5 × 2 mm section in the papillary muscles. **v**_**1**_ tracks reveal that cells are aligned along the length of the papillaries, while **v**_**2**_ and **v**_**3**_ tracks are indicative of a concentric sheetlet arrangment. **(c)** Digitally bisected heart viewed from the LV anterior wall. **(d)** Magnified view of tracks in a 2.5 × 1 × 1 mm section in the LV septal wall. The transmural variation in dominant cell orientation can be seen from the **v**_**1**_ tracks. This relatively gradual variation in cell orientation belies sharp discontinuities in sheetlet orientation as seen in **v**_**3**_ tracks. Dashed lines overlaid on **v**_**3**_ tracks approximate the underlying sheetlet laminae. **(e)** Map of clusters based on orientation of **v**_**3**_; **v**_**1**_ tracks intersecting sample clusters 13, 18 and 22 illustrates distinct cell alignment in discretised sheetlet structures.

Tracks were reconstructed in the whole heart based on **v**_**2**_ and **v**_**3**_ (Fig. 3a–d). To visualise tracks in the myocardial wall, hearts were digitally bisected. The data quality is reflected in the length and coherence of **v**_**2**_ and **v**_**3**_tracks. In the papillary muscles, tracks based on **v**_**1**_suggest that cells are oriented in a linear manner (Fig. 3a,b). Tracks based on **v**_**2**_and **v**_**3**_additionally point to a concentric arrangment of cell layers, as seen by the circumferential and radial orientation of **v**_**2**_and **v**_**3**_tracks respectively, and a twisting of the papillary muscle along its length.

We found, unsurprisingly, that **v**_**1**_ tracks reconfirm the transition from left- to right-handed helical fibres progressing from the LV subepicardium to subendocardium. **v**_**3**_tracks were largely oriented in a radial manner in the LV anterior and posterior walls, suggesting that in these regions, myocardial sheetlets in relaxed hearts are aligned in planes that deviate only little from the myocardial wall surface^8^. Despite a smooth transition in **v**_**1**_ track orientations over much of the heart, sharp discontinuities are evident in sheetlet and sheetlet-normal orientations particularly in the LV lateral and septal walls, including V-, N- and herringbone-shaped sheetlet arrangements (Fig. 3c,d), as previously demonstrated in histological studies^8^,^22^.

Clustering of tracks was performed based on **v**_**3**_orientation, and clusters in a single mid-coronal slice are shown (Fig. 3e). Tracks based on **v**_**1**_ were then propagated from three sample regions (13, 18 and 22), illustrating the primary cell orientations in discretised sheetlet structures. The reconstructed **v**_**1**_ tracks are highly organised and have distinctly different anatomical distributions dependent on the segmentation of **v**_**3**_.

The 95% COU in **v**_**1**_, **v**_**2**_ and **v**_**3**,_ averaged across the global myocardium, were 3.7° ± 0.2°, 10.9° ± 0.4°, and 10.6° ± 0.5°, signifying excellent precision in measurements (mean ± standard deviation; N = 5 hearts).

### Quantitative mapping

**Figure 4:**
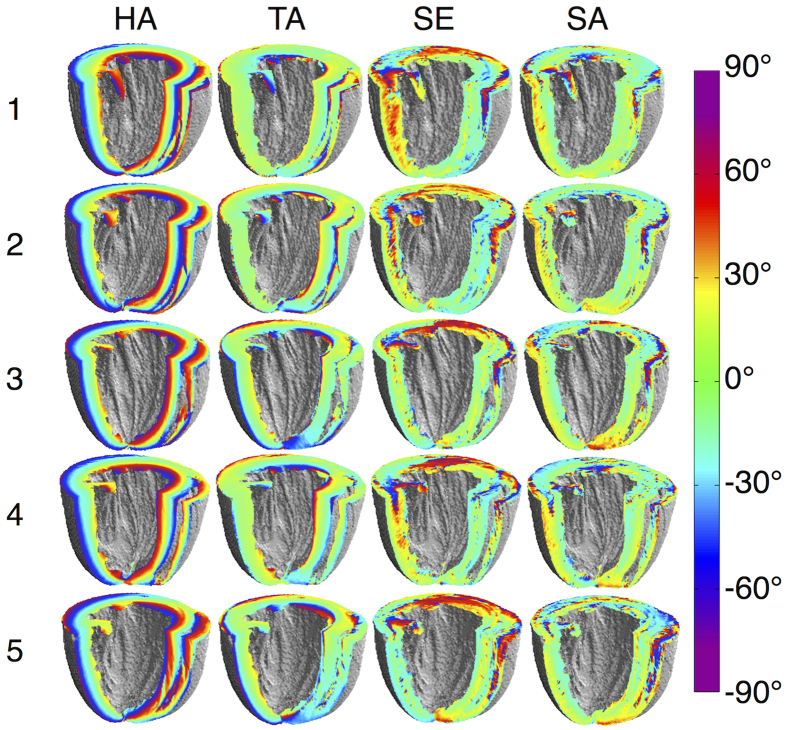
Quantitative angle maps in five healthy *ex vivo* rat hearts. Both cell and sheetlet orientations are well preserved over all hearts. The transmural transition from left to right helical arrangement of cells is reflected in the helix angle (HA) maps. The transverse angle (TA) remains low in the majority of the myocardium, signifying a primarily circumferential arrangement of cells when projected onto the short-axis plane. The sheet azimuth (SA) is similarly characterised by small angles. This indicates that the projections of the sheetlet-normal onto short-axis plane are predominantly radial. In contrast, the sheet elevation (SE) maps show that the sheetlet-normal varies regionally. In the basal slice as shown for example, SE is near 0° in the LV lateral and septal walls, and varies between approximately 60° to 0° from the LV anterior wall subepicardium to subendocardium. A custom cyclic colour map was used to avoid discontinuities at transitions between −90° and +90°.

Maps of global myocardial helix angle (HA), transverse angle (TA), sheet elevation (SE) and sheet azimuth (SA), as defined in *Methods*, are presented in Fig. 4. The maps are generally consistent across the five hearts. In all hearts, HA maps point to a left to right helical arrangement of cells from the LV subepicardium to subendocardium. TA maps are predominantly close to 0°, reflecting a circumferential arrangement of cells when projected onto the local short-axis plane. We observe that SA maps were remarkably consistent and generally close to 0°. This suggests that sheetlets are organised in a concentric manner with respect to the local short-axis plane. There is greater heterogeneity in SE maps, consistent with observed discontinuities in the **v**_**3**_tracking data.

**Figure 5:**
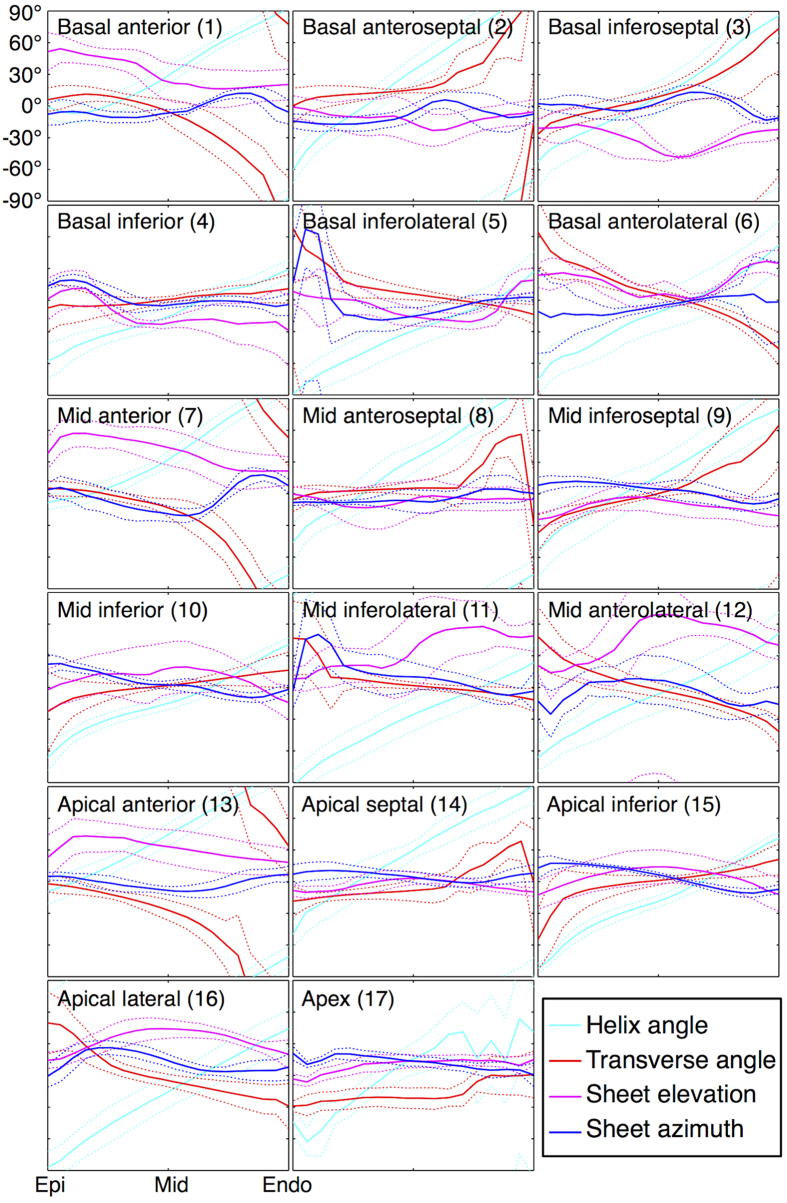
Transmural variation in helix angle, transverse angle, sheet elevation and sheet azimuth. Regions were defined by the 17-segment AHA model. Means (solid lines) and standard deviations (dotted lines) over the five hearts are given. Angles were normalised to wall thickness in each region.

Transmural profiles of HA, TA, SE and SA as measured in 17 segments in the LV, based on the American Heart Association (AHA) heart model^23^, are presented (Fig. 5) and their linearity and ranges summarised (Table 1). The transmural profiles of HA were found to be linear across AHA1-16 (R^2^ ≥ 0.95). However, there was a broad mean transmural range of between 83.8° in the basal inferior region to 163° in the basal anteroseptal region. While |TA| appeared relatively low in the majority of the myocardium, there were distinct negative deviations towards the subendocardium in the apical to basal anterior wall, and positive deviations towards the subendocardium in the mid and basal septum. This resulted in a lower linearity (0.55 ≤ R^2^ ≤ 0.94) as compared to the HA, and a mean transmural range of between −125° in the mid anterior wall and 92.1° in the mid inferoseptal wall. Among the four angles, SA was the closest to 0° across the LV, and had a range spanning −50.6° in the mid inferolateral wall to 40.9° in the basal anterolateral wall. Localised peaks in SA were observed near the subepicardium in the mid and basal inferolateral walls. While |SE| varied widely, SE had the most stable range of between −42.5° in the basal anterior wall and 15.6° at the apex, indicating that the elevation of the sheetlet laminae in the local radial plane is similar in the subendocardium and subepicardium within the same region. Here, “apex” refers to the apical region as defined by region 17 in the 17-segment AHA heart model. Linearity of SE and SA are both highly variable (0.02 ≤ R^2^ ≤ 0.86) and consistent with the heterogeneous and discontinuous nature of sheetlet arrangements.

**Figure 6:**
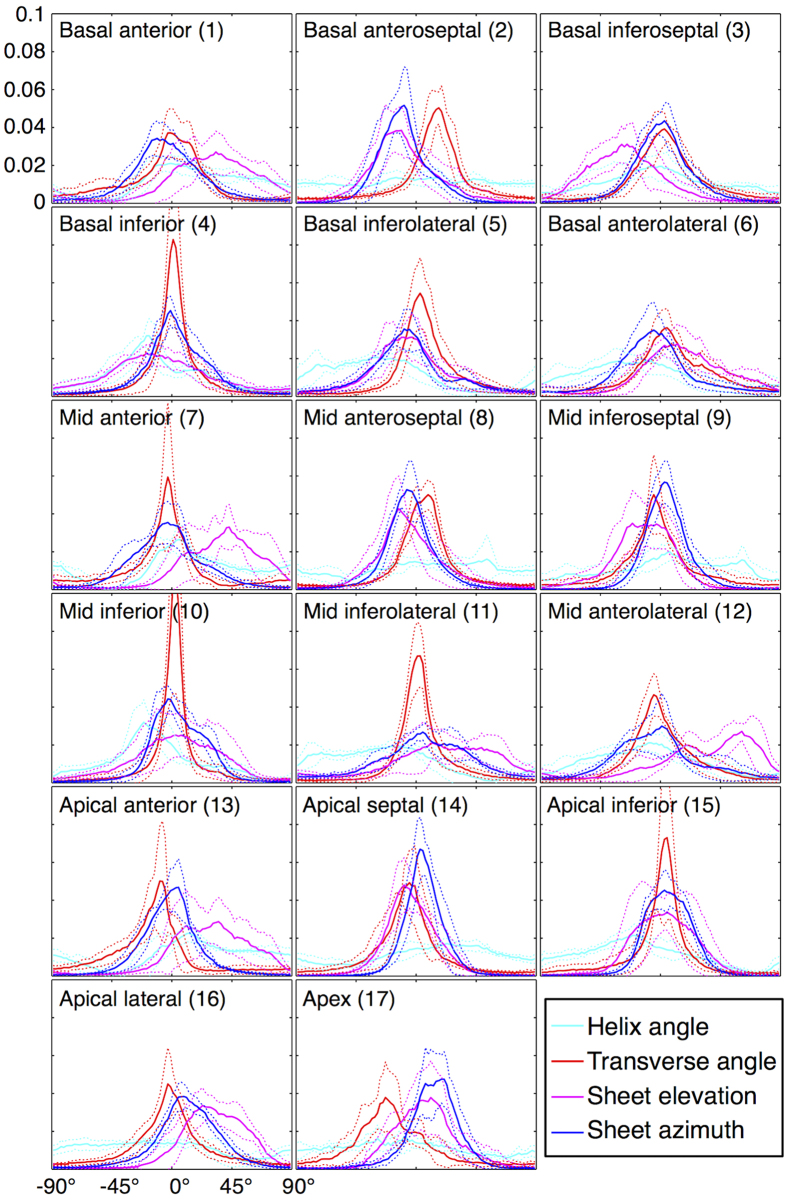
Histograms of the helix angle, transverse angle, sheet elevation and sheet azimuth. Regions were defined by the 17-segment AHA model. Means (solid lines) and standard deviations (dotted lines) over the five hearts are given.

Histograms of HA, TA, SE and SA describe the angular distribution in the 17 AHA segments (Fig. 6). HA were broadly distributed across all segments due to its linear transmural profile. TA exhibited the sharpest peaks near 0° in a number of segments, with positive skew observed in the mid and basal lateral walls and negative skew in the apical to basal anterior wall. Of note is the −25° peak shift in TA in AHA17 that reflects the clockwise spiral of cells towards the apex. Sheetlets were generally radially oriented within the local short-axis plane, with −11.6° ≤ max(SA) ≤ 22.1°. In contrast, there was greater variation in sheetlet elevation with respect to the local short-axis plane with −24.2° ≤ max(SE) ≤ 58.7°. SE was more broadly distributed than SA and had a bimodal distribution in the mid anterolateral wall. Supporting research data are available upon request.

## Discussion

Previous studies had reported modest improvements in SNR using dynamic receiver gains, including a 10% increase in SNR in T_1_-weighted imaging^24^. We show its greater impact on imaging sequences with inherently low SNR such as DTI, and report an 85% improvement in SNR in the DW data. This led to an increase in eigenvector precision, reflected by a 41% reduction in 95% COU. The dynamic gain setting mitigates accumulation of noise between the pre-amplifier and the analogue-to-digital converter, and minimises rounding errors of the A/D converter which may increase noise. The choice of receiver gain is primarily governed by b-value. For experiments with multiple b-values, optimal results are obtained with multiple gain settings. We observed that the precision of estimates was not greatly affected by the simulated poor gradient calibration as the bias in diffusion-weighting varies smoothly over 3D space. However, the FA in the buffer with isotropic diffusion was artificially high. Rather than acquire new calibration data for each DW sequence^25^,^26^, our modified method need only be performed once, is non-sequence specific and improves both diffusion and geometric accuracy. The precision in eigenvector estimates are compromised when the effect of imaging gradients and cross-terms are ignored. However, they are comparable using both reversed diffusion gradient correction and prospective adjustment methods. Our implementation, improved eigenvector precision without additional scan time overhead. In practice, the 95% COU, and the effect of imaging gradients and cross-terms, were lowered further as crushers were minimised such that b_imaging_ = 12 s/mm^2^. Sample heating affects water diffusion, and a 1 °C increase in temperature corresponds to a 2.4% increase in diffusion coefficient^27^. Heating can be avoided by using longer repetition times, fewer echoes and lower b-values, at the cost of acquisition time, signal efficiency and diffusion contrast. Failure to account for sample heating over the imaging timecourse risks systematically biasing the DTI data. For instance, when scans are not interleaved, DW scans may be acquired at up to 3.6 °C higher temperature than non-DW scans. Here we applied initial warm-up DW scans and interleaved DW and non-DW scans by image volumes, and mitigated temperature fluctuations to within 1 °C. We found that 8 warm-up scans at b = 1,000 s/mm^2^ were required to bring the sample to thermal steady state. However, the additional 1:46 h required was prohibitive, and we substituted this with a 16 min warm-up scan at b = 10,000 s/mm^2^. Heating of the gradient system also influences the magnetic field and may lead to quantification errors, as shown in phase contrast MRI^28^. Further work is needed to quantify the effect size of gradient system heating on diffusion MRI measurements, and to develop correction strategies.

Recent *ex vivo* DTI studies have acquired 3D data at isotropic resolutions up to 200 μm in rats^21^ and and 43 μm in mice^29^. However, in the latter study, the hearts were not rigidly embedded, and sample motion between scans mandated non-rigid registration and exclusion of a number of basal slices due to mis-registration. In addition, only the minimum of 6 DW directions were sampled, and therefore reproducibility of eigenvector estimates would have been compromised^30^. Concerns of reproducibility of **v**_**2**_ and **v**_**3**_ in general^21^ warrant wider reporting of eigenvector error metrics. Furthermore, angle maps are rarely reported at the apex due to challenges in acquisition and quantification, and are often unreliable^31^. Our refinements coupled with robust implementation of local coordinate systems facilitated estimation of angle maps near the apex with much greater consistency across hearts, thereby improving the prospect of building whole heart atlases for modelling cardiac structure^31^.

In this study, we obtained robust DTI measurements at an isotropic resolution of 100 μm, where each voxel contains on the order of tens of myoctes. The acquisition can be accelerated, by using strategies such as compressed sensing^32^, and the resultant time savings reinvested in improving spatial resolution. Even so, this resolution invariably falls short of microscopy methods. However, in various fields of study, for example in structure-based electro-mechanical modelling, it is essential to preserve the global 3D cell and sheetlet architecture and the resolution may only need to be high enough to distinguish and represent functionally distinct groups of myocytes. For such purposes, in particular for characterisation of large sample numbers, high-resolution *ex vivo* DTI may serve as the method of choice.

Eigenvector tracking in the heart provides a powerful means to visualise cell and sheetlet orientations in the whole heart as inferred from DTI, beyond just individual tensors and glyphs. For example, we inferred from **v**_**2**_and **v**_**3**_tracks, that there was a concentric organisation of cell layers and a lengthwise twisting in the papillary muscles. These observations have important functional ramifications; the action of the papillary muscles may not be limited to contraction along its length, but may involve shear and torsion as informed by sheetlet and sheetlet-normal orientations. Clustering of tracks is a nascent way to classify myocyte organisation into supra-cellular morphological groupings. This will inform structure-based cardiac models and improve region-specific quantitative assessments of remodelling. An early study employed K-means clustering to classify **v**_**1**_ tracks^33^. However, as the myocardium demonstrates orthotropic properties, it is anticipated that grouping according to **v**_**2**_ and **v**_**3**_ would improve the accuracy, reliability and physiological significance of clustering. While not suggesting that these clusters hint at the presence of discrete muscle bundles in the heart, a notion rightly dismissed^3^, we propose that there is sufficient evidence by way of distinctly oriented and abutting groups of myocytes both from our study and others^4^,^5^ to support extensions of continuum approaches to modelling cardiac structure and function^3^.

Mapping of tissue architecture is fundamental to understanding the contractile dynamics of the heart. While methods for *in vivo* diffusion MRI continue to improve, they remain resolution-limited and prone to artefacts arising from motion and strain. Our refinements address a series of technical hurdles and facilitate resolution of fine anatomical structures and detection of myocardial sheetlet structure in preclinical small animal imaging *ex vivo* at an unprecedented level of detail. These techniques are equally applicable in human *ex vivo* studies^34^,^35^. They can expand the role of high-resolution *ex vivo* DTI, and open up new research opportunities in atrial, valvular and vascular disease. Robust detection of sheetlet structures offers a new dimension for segmentation, tracking, and modelling of cardiac histo-anatomy, to further our understanding of individual 3D architectures of the heart. These insights will help to refine electro-mechanical models of the heart, which can bridge the gap between dynamic measurements of morphological features *in vivo* and high-resolution characterisation of microstructure *ex vivo*, paving the way towards patient-specific models of cardiac biomechanics^15^.

## Methods

### Sample preparation

Experimental investigations conformed to the UK Home Office guidance on the Operations of Animals (Scientific Procedures) Act 1986 and were approved by the University of Oxford ethical review board. Five hearts were excised from Sprague-Dawley rats, weighing between 199 and 221 g, during terminal anaesthesia. Isolated hearts were swiftly perfused in Langendorff constant pressure mode at 80 mmHg with oxygenated (95% O_2_/5% CO_2_) modified Krebs-Henseleit solution (in [mM]: NaCl 118.5, NaHCO_3_ 25.0, KCl 4.75, MgSO_4_ 1.22, KH_2_PO_4_ 1.21, Glucose 11.0, CaCl_2_ 1.84), cardioplegically arrested with high potassium (in [mM]: NaCl 125.0, KCl 20.0, MgCl_2_ 1.0, HEPES 5.0, Glucose 11.0, CaCl_2_ 1.8) and perfused with low osmolality Karnovsky’s fixative (300 ± 10 mOsm; in [%]: PFA 0.45, glutaraldehyde 0.57, sodium cacodylate 0.97) doped with 2 mM gadolinium (Gd) complex Prohance (Bracco, MN, USA). The hearts were then immersed in 50 mL of the same fixative and kept at 4 °C for a minimum of 14 days to allow complete fixation. The fixative was changed on day 3. The fixative and contrast agent were specified to minimise osmotic gradients and shortening of T_2_. Prior to imaging, samples were rinsed three times in PBS + 2 mM Gd, and embedded in 1% agarose gel (Web Scientific, Crewe, UK) in PBS + 2 mM Gd to avoid sample motion and gradients in osmolality and contrast agent concentration.

### Data acquisition

Non-selective 3D fast spin echo DTI data were acquired on a 9.4 T preclinical MRI scanner (Agilent, CA, USA) with a shielded gradient system (max gradient strength = 1 T/m, rise time = 130 μs), and transmit/receive birdcage coil (inner diameter = 20 mm; Rapid Biomedical, Rimpar, Germany). Acquisition parameters were: repetition time = 250, echo time = 9.3 ms, echo spacing = 4.9 ms, echo train length = 8, field-of-view = 20 × 16 × 16 mm, resolution = 100 × 100 × 100 μm, number of non-DW images = 8, number of DW directions = 61^36^, b_effective_ = 1,000 s/mm^2^, diffusion duration (δ) = 2 ms, diffusion time (Δ) = 5.5 ms, receiver bandwidth = 100 kHz, acquisition time = 15 h 20 min. Warm-up DW scans were performed prior to the DTI acquisition to bring the system towards a thermal steady state, with b = 10,000 s/mm^2^ and acquisition time = 16 min. Noise data were acquired with the same sequence with TR = 67 ms and without radiofrequency pulses. Anatomical MRI data of the identical imaging volume were acquired at an isotropic resolution of 33 μm with a spoiled gradient echo sequence^37^. One heart was re-scanned in a separate session to assess the effects of technical refinements as follows.

### Dynamic receiver gain

The receiver gain was manually specified for non-DW and DW scans, to maximise but not exceed the dynamic range of the analogue-to-digital converter. Here, the receiver gain was identical across scans with different DW directions, based on the assumption that the overall signal intensities across different DW directions would be broadly similar, given the wide range of cell orientations in 3D across the heart. DTI data were acquired in one heart with receiver gain for non-DW/DW scans set to 2/2 dB (static gain) and 2/14 dB (dynamic gain). Additional non-localised gradient echo data were acquired at the different receiver gain settings with 16 averages, and the respective mean peak signals were used to normalize the DTI data. SNR was measured in the whole myocardium in each non-DW and DW scan with static and dynamic gain. DTI was performed with both gain settings, and the 95% COU in eigenvectors **v**_**1**_, **v**_**2**_ and **v**_**3**_ were reported.

### Gradient system calibration

As the applied b-value depends on the square of the gradient amplitude, calibration of diffusion measurements in a reference phantom with known diffusivity offers a quick, sensitive and accurate method for gradient calibration^19^. This calibration method was performed prior to the high resolution DTI experiments. Briefly, this involved scanning a 20 mm glass tube filled with 99% cyclooctane (Sigma-Aldrich, MO, USA) with a 2D echo planar imaging sequence while acquiring sample temperature data. Measured diffusivities in x-, y- and z-directions were compared to reference diffusivity data^38^ and a correction factor for the linear gradient scaling in each orthogonal direction was calculated and applied. For comparison, a poorly calibrated gradient system was simulated by reducing the gradient scaling in the y- and z-directions by 10%. DTI was performed in both calibrated and offset settings and the FA and 95% COU were reported.

### Prospective diffusion gradient adjustment

The exact diffusion-weighting applied, as defined by the effective **b** matrix, includes contributions from the diffusion gradients, imaging gradients and their interaction with each other, where **b**_effective_ = **b**_diffusion _+ **b**_imaging _+ **b**_cross-terms_. However, the contributions from cross-terms are often ignored as a first approximation. As the strength and duration of the imaging gradients is increased, as is often required for crushing of undesired signals, so does their contribution to the effective b-value. To illustrate this, the refocusing crusher gradient strength and duration were increased such that b_imaging_ = 61 s/mm^2^. One established method for correction involves acquiring a 2^nd^ DW dataset with reversed diffusion gradient polarity^39^, however, this entails doubling the acquisition time. To avoid the additional scan burden, while compensating for variable b in uncorrected scans, we adjusted the diffusion gradient strength, G, prospectively in each DW acquisition such that b_effective_ = b_nominal_. A similar approach was adopted by Lundell, *et al*.^40^. **b**_effective_ was calculated numerically on the scanner, and G was updated iteratively until |b_effective_ − b_nominal_| < 0.001 *b_nominal_. DTI was performed (i) without correction, (ii) with reversed diffusion gradient correction and (iii) with prospective diffusion gradient adjustment. The effective b-value in each DW direction was reported, together with the 95% COU of the principal eigenvectors.

### Interleaving DW and non-DW scans

Heating in *ex vivo* samples occur due to (i) absorption of radiofrequency energy, (ii) heat transfer from the gradient system that warms during the application of strong diffusion gradients, and (iii) lack of homeostatic capacity. Higher temperatures raise tissue diffusivities and can shorten T_2_^41^, leading to lower signal intensities in DW and non-DW scans. To mitigate temperature fluctuations over the imaging timecourse, DW and non-DW scans were interleaved. To illustrate the magnitude of temperature fluctuations and their effect on the acquired images, single non-DW datasets were acquired immediately following warm-up scans comprising 0 to 8 DW scans. Each DW scan was performed with b = 1,000 s/mm^2^ and required 13.5 min. A 30 min pause between each warm-up and non-DW scan cycle was introduced to allow for sample cooling. Temperature was measured using a thermistor embedded in epoxy resin. The calibrated thermistor was connected to a Harvard Apparatus homeostatic temperature control unit (Harvard Apparatus, Kent, UK) and secured to the surface of the sample tube. Temperature was recorded at 1 Hz on a Powerlab/30 with Chart v5.0 (AD Instruments, Bella Vista, Australia) and smoothed with a 60 s sliding window. The mean signal intensity in the global myocardium was measured in each non-DW image, and normalised to that of the non-DW image without prior warm-up scans.

### Data analysis

Data from DTI experiments were fitted to a 3D tensor using non-linear least squares. The 8 non-DW volumes and the 61 DW volumes were fitted simultaneously. The mean apparent diffusion coefficient (ADC) and FA were calculated using in-house code (Matlab2013a, Natick, MA, USA). SNR was calculated by dividing the mean signal in whole myocardium by the standard deviation of the noise data at the respective gain setting^42^. To address eigenvector sorting issues^2^,^21^, and quantify the error in **v**_**1**_, **v**_**2**_, **v**_**3**_, wild bootstrapping was performed. Here, diffusion tensors were first fitted to the DTI data using non-linear least squares. The sign of the residuals across the imaging volumes with different DW directions were permuted randomly, following the Rademacher distribution. The diffusion tensor model was fitted to this modified signal. This process was repeated to generate a probability distribution comprising 1000 tensor volumes, from which 95% COU results are reported in the whole myocardium^43^. The myocardium was defined by thresholding the signal intensity in the b = 0 images, mean ADC and FA maps, as normalised to their respective global maxima: (0.1 < b = 0 signal <1) 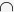 (0 < mean ADC < 0.6) 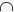 (0.1 < FA < 1). Regions corresponding to λ_2_/λ_3 _< 1.05, i.e. where sheetlet structures are poorly defined, were excluded. These regions were sparsely and well distributed across the myocardium, and constituted 0.116 ± 0.008 of the total cardiac volume (mean ± standard deviation; N = 5 hearts).

**Figure 7:**
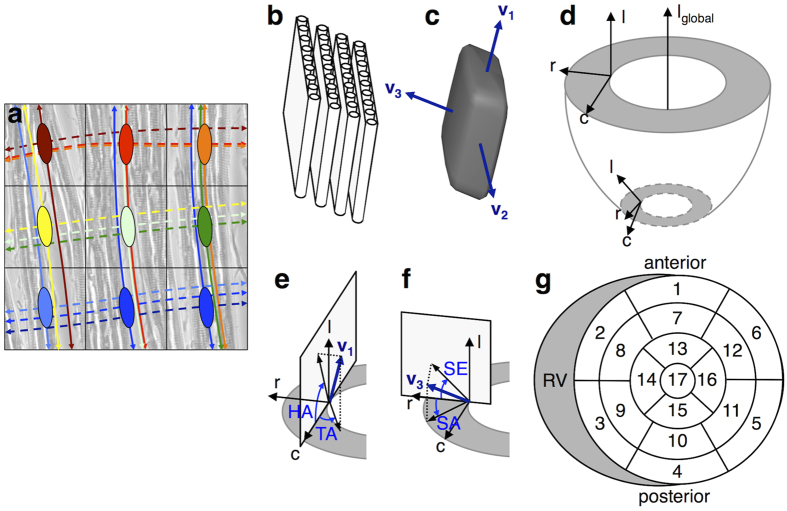
Methods and terminology. **(a)** Exemplar tensors overlaid on a bright-field microscopy image of *ex vivo* rat myocardium in a 3 × 3 voxel neighbourhood. Each of nine tensors is represented in a different colour. Tracks are propagated in matching colours from individual seed voxels, based on **v**_**1**_ (solid lines) and **v**_**3**_ (dashed lines). **v**_**1**_ tracks correspond to the fibre long axis, and **v**_**3**_ tracks correspond to sheetlet-normal directions. Tracks based on **v**_**2**_ are not shown. While each 100 μm voxel contains a single tensor, voxels can be traversed by multiple tracks, originating from other seed voxels. **(b)** Schematic of cardiomyocytes arranged in four sheetlet layers. **(c)** Superquadric glyph describing the diffusion tensor and principal eigenvectors **v**_**1**_, **v**_**2**_ and **v**_**3**_ in a volume containing cardiomyocytes in (**b**). **(d)** Orientations of the longitudinal, radial and circumferential axes (l, r, and c) in the local coordinate system depend on the surface curvature of the heart, shown here in a basal and an apical slice. Local radial vectors (**r**) were computed in the LV using Laplace’s method^50^. Local circumferential vectors (**c**) were defined as vectors perpendicular to local radial and global longitudinal (**l**_**global**_) unit vectors, where the global longitudinal vector was determined by linear fitting of the centres-of-mass of the segmented LV cavity in 2D short-axis planes. Local longitudinal vectors (**l**) were defined as vectors perpendicular to local radial and circumferential unit vectors. Short-axis, tangential and radial planes correspond to the c-r, c-l and l-r planes respectively. **(e)** Helix angle (HA) is the angle subtended by the projection of **v**_**1**_onto the tangential plane and the short-axis plane. Transverse angle (TA) is the angle subtended by the projection of **v**_**1**_onto the short-axis plane and the local circumferential axis. **(f)** Sheet elevation (SE) is the angle subtended by the projection of **v**_**3**_ onto the radial plane and the short-axis plane. Sheet azimuth (SA) is the angle subtended by the projection of **v**_**3**_onto the short-axis plane and the local radial axis. **(g)** The 17-segment AHA segmentation of myocardial regions^23^.

Whole heart eigenvector tracking was performed along **v**_**1**_, **v**_**2**_, and **v**_**3**_ using Diffusion Toolkit and Trackvis^44^. Tracking was based on the 2^nd^ order Runge Kutta method^45^. This deterministic approach enabled propagation of streamlines at sub-voxel resolution (Fig. 7a), and reconstruction of extensive tracks (see Supplementary Videos 1 and 2). Segmentation of functional groups of voxels was performed using mean-shift clustering on the SE and SA maps as follows: phase unwrapping was performed by mapping A* = *e*^*2Ai*^, where *A* denotes the angle in radians. In total, six features were passed to the clustering algorithm; two for the unwrapped angle maps, three for the spatial location (x, y, z), and one for normalised transmural depth. A flat kernel was used for mean-shift clustering^46^. The spatial features, angle maps and transmual depth had bandwidths of 10 mm, 0.3°, and 10% respectively. Regions with a volume smaller than 0.1 mm^3^ were merged with their nearest neighbour. Following clustering and merging, a 3^3^ mode filter was applied. Tracks based on **v**_**1**_were propagated from 3 sample clusters in the LV septal wall.

A schematic of the arrangement of cardiomyocytes into sheetlets is presented in Fig. 7b. The orientation of these cells can be estimated with DTI and represented by a superquadric glyph^47^ as described by the principal eigenvectors (Fig. 7c). Local coordinate systems (Fig. 7d) were used to define HA, TA, SE and SA (Fig. 7e,f). SE has previously been referred to as intersection angle^7^, sheet angle^48^ and tertiary eigenvector inclination angle^49^, however, as the nomenclature for **v**_**3**_orientation is poorly defined, we have used SE and SA, which we feel are better descriptors of the angles of concern. Global maps of HA, TA, SE and SA in the myocardium are presented. The 17-segment AHA segmentation^23^ of LV myocardial regions was used for regional quantification of angles (Fig. 7g). The transmural profile of HA, TA, SE and SA was calculated and normalized across the LV wall thickness on a regional basis, ignoring the few voxels along the global longitudinal axis at the apex where the local coordinate system is degenerate. The linearities (R^2^) and ranges of these transmural profiles were determined. Histograms of HA, TA, SE and SA in each region are presented.

## Additional Information

**How to cite this article**: Teh, I. *et al*. Resolving Fine Cardiac Structures in Rats with High-Resolution Diffusion Tensor Imaging. *Sci. Rep.* **6**, 30573; doi: 10.1038/srep30573 (2016).

## Supplementary Material

Supplementary Information

Supplementary Video S1

Supplementary Video S2

## Figures and Tables

**Table 1:** Linearity and range of transmural helix angle, transverse angle, sheet elevation and sheet azimuth.

	Helix angle		Transverse angle		Sheet elevation		Sheet azimuth	
	Linearity	Range (°)	Linearity	Range (°)	Linearity	Range (°)	Linearity	Range (°)
1	0.95 ± 0.03	109.1 ± 9.2	0.68 ± 0.39	−85.2 ± 54	0.80 ± 0.07	−42.5 ± 11	0.33 ± 0.32	16.6 ± 12
2	1.00 ± 0.00	162.5 ± 5.1	0.55 ± 0.31	75.8 ± 48	0.18 ± 0.16	−6.9 ± 9.8	0.27 ± 0.27	14.7 ± 13
3	0.98 ± 0.02	133.2 ± 5.0	0.92 ± 0.04	91.9 ± 35	0.15 ± 0.13	−11.3 ± 14	0.13 ± 0.10	−3.2 ± 11
4	0.99 ± 0.01	83.8 ± 7.3	0.86 ± 0.14	18.6 ± 32	0.52 ± 0.37	−33.1 ± 16	0.52 ± 0.29	−21.3 ± 6.5
5	0.99 ± 0.01	104.1 ± 4.8	0.68 ± 0.31	−59.7 ± 17	0.11 ± 0.12	−2.3 ± 18	0.47 ± 0.32	12.8 ± 58
6	0.99 ± 0.00	114.4 ± 13	0.93 ± 0.04	−89.8 ± 16	0.08 ± 0.04	5.8 ± 11	0.44 ± 0.41	40.9 ± 45
7	0.97 ± 0.01	120.4 ± 5.2	0.80 ± 0.16	−124.7 ± 18	0.74 ± 0.06	−39.4 ± 7.7	0.15 ± 0.08	12.6 ± 14
8	0.99 ± 0.00	145.4 ± 4.5	0.49 ± 0.37	59.7 ± 42	0.08 ± 0.08	2.3 ± 6.3	0.49 ± 0.33	13.0 ± 12
9	0.99 ± 0.01	129.7 ± 7.3	0.91 ± 0.04	92.1 ± 38	0.13 ± 0.20	−0.6 ± 7.5	0.69 ± 0.17	−21.8 ± 7.0
10	0.98 ± 0.01	94.7 ± 2.5	0.73 ± 0.39	34.1 ± 41	0.13 ± 0.15	−9.5 ± 9.7	0.78 ± 0.20	−31.6 ± 9.7
11	0.99 ± 0.00	109.7 ± 6.6	0.59 ± 0.34	−10.9 ± 79	0.80 ± 0.28	2.4 ± 99	0.47 ± 0.26	−50.6 ± 36
12	0.99 ± 0.01	107.9 ± 8.9	0.94 ± 0.02	−72.9 ± 17	0.54 ± 0.32	−7.1 ± 97	0.22 ± 0.38	−8.5 ± 26
13	0.99 ± 0.01	121.8 ± 9.0	0.71 ± 0.37	−101.6 ± 69	0.52 ± 0.35	−21.3 ± 17	0.20 ± 0.12	−0.3 ± 12
14	0.98 ± 0.01	131.5 ± 11	0.59 ± 0.32	53.1 ± 25	0.08 ± 0.10	1.8 ± 4.7	0.26 ± 0.12	−7.9 ± 4.3
15	0.98 ± 0.01	112.6 ± 5.7	0.62 ± 0.34	49.2 ± 42	0.02 ± 0.03	0.8 ± 3.6	0.86 ± 0.12	−32.6 ± 6.5
16	0.99 ± 0.00	130.5 ± 8.4	0.80 ± 0.16	−72.4 ± 20	0.07 ± 0.05	6.3 ± 7.1	0.33 ± 0.29	−14.5 ± 14
17	0.76 ± 0.36	120.9 ± 56	0.57 ± 0.26	27.3 ± 18	0.44 ± 0.28	15.6 ± 10	0.55 ± 0.22	−16.0 ± 11

Regions were defined by the 17-segment AHA model. Linearity and range were measured from the LV subepicardium to subendocardium. Means and standard deviations are reported over five hearts.

## References

[b1] Streeter D. D. Jr In Handbook of Physiology, Section 2: The Cardiovascular System 61–112 (Williams and Wilkins, 1979).

[b2] Gilbert S. H., Benson A. P., Li P. & Holden A. V. Regional localisation of left ventricular sheet structure: integration with current models of cardiac fibre, sheet and band structure. European journal of cardio-thoracic surgery: official journal of the European Association for Cardio-thoracic Surgery 32, 231–249, 10.1016/j.ejcts.2007.03.032 (2007). 17462906

[b3] Anderson R. H., Ho S. Y., Redmann K., Sanchez-Quintana D. & Lunkenheimer P. P. The anatomical arrangement of the myocardial cells making up the ventricular mass. European journal of cardio-thoracic surgery: official journal of the European Association for Cardio-thoracic Surgery 28, 517–525, 10.1016/j.ejcts.2005.06.043 (2005). 16179192

[b4] Costa K. D., Takayama Y., McCulloch A. D. & Covell J. W. Laminar fiber architecture and three-dimensional systolic mechanics in canine ventricular myocardium. American journal of physiology. Heart and circulatory physiology 276, H595–H607 (1999). 10.1152/ajpheart.1999.276.2.H595 9950861

[b5] Dokos S., Smaill B. H., Young A. A. & LeGrice I. J. Shear properties of passive ventricular myocardium. *American journal of physiology*. Heart and circulatory physiology 283, H2650–2659, 10.1152/ajpheart.00111.2002 (2002). 12427603

[b6] Harrington K. B. . Direct measurement of transmural laminar architecture in the anterolateral wall of the ovine left ventricle: new implications for wall thickening mechanics. American journal of physiology. Heart and circulatory physiology 288, H1324–1330, 10.1152/ajpheart.00813.2004 (2005). 15550521 PMC2822837

[b7] Helm P. A., Tseng H. J., Younes L., McVeigh E. R. & Winslow R. L. *Ex vivo* 3D diffusion tensor imaging and quantification of cardiac laminar structure. Magnetic resonance in medicine: official journal of the Society of Magnetic Resonance in Medicine/Society of Magnetic Resonance in Medicine 54, 850–859, 10.1002/mrm.20622 (2005). PMC2396270 16149057

[b8] Hales P. W. . Histo-anatomical structure of the living isolated rat heart in two contraction states assessed by diffusion tensor MRI. Progress in biophysics and molecular biology 110, 319–330, 10.1016/j.pbiomolbio.2012.07.014 (2012). 23043978 PMC3526796

[b9] Burton R. A. . Three-dimensional histology: tools and application to quantitative assessment of cell-type distribution in rabbit heart. Europace: European pacing, arrhythmias, and cardiac electrophysiology: journal of the working groups on cardiac pacing, arrhythmias, and cardiac cellular electrophysiology of the European Society of Cardiology 16 Suppl 4, iv86–iv95, 10.1093/europace/euu234 (2014). PMC4217519 25362175

[b10] Basser P. J., Mattiello J. & LeBihan D. MR diffusion tensor spectroscopy and imaging. Biophysical journal 66, 259–267, 10.1016/S0006-3495(94)80775-1 (1994). 8130344 PMC1275686

[b11] Scollan D. F., Holmes A., Winslow R. & Forder J. Histological validation of myocardial microstructure obtained from diffusion tensor magnetic resonance imaging. The American journal of physiology 275, H2308–2318 (1998). 9843833 10.1152/ajpheart.1998.275.6.H2308

[b12] Hsu E. W., Muzikant A. L., Matulevicius S. A., Penland R. C. & Henriquez C. S. Magnetic resonance myocardial fiber-orientation mapping with direct histological correlation. The American journal of physiology 274, H1627–1634 (1998). 9612373 10.1152/ajpheart.1998.274.5.H1627

[b13] Kung G. L. . The presence of two local myocardial sheet populations confirmed by diffusion tensor MRI and histological validation. J Magn Reson Imag 34, 1080–1091, 10.1002/jmri.22725 (2011). PMC3195899 21932362

[b14] Nordbo O. . A computational pipeline for quantification of mouse myocardial stiffness parameters. Computers in biology and medicine 53, 65–75, 10.1016/j.compbiomed.2014.07.013 (2014). 25129018 PMC4625543

[b15] Krishnamurthy A. . Patient-Specific Models of Cardiac Biomechanics. Journal of computational physics 244, 4–21, 10.1016/j.jcp.2012.09.015 (2013). 23729839 PMC3667962

[b16] Gomez A. D., Bull D. A. & Hsu E. W. Finite-Element Extrapolation of Myocardial Structure Alterations Across the Cardiac Cycle in Rats. J Biomech Eng-T Asme 137, Artn 10.1115/1.4031419 (2015). PMC4844231 26299478

[b17] Choi Y. J., Constantino J., Vedula V., Trayanova N. & Mittal R. A New MRI-Based Model of Heart Function with Coupled Hemodynamics and Application to Normal and Diseased Canine Left Ventricles. Frontiers in bioengineering and biotechnology 3, 140, 10.3389/fbioe.2015.00140 (2015). 26442254 PMC4585083

[b18] Mukherjee P., Chung S. W., Berman J. I., Hess C. P. & Henry R. G. Diffusion tensor MR imaging and fiber tractography: technical considerations. AJNR. American journal of neuroradiology 29, 843–852, 10.3174/ajnr.A1052 (2008). 18339719 PMC8128579

[b19] Teh I., Maguire M. L. & Schneider J. E. Efficient gradient calibration based on diffusion MRI. Magnetic resonance in medicine: official journal of the Society of Magnetic Resonance in Medicine/Society of Magnetic Resonance in Medicine, 10.1002/mrm.26105 (2016). PMC5217059 26749277

[b20] Thelwall P. E., Shepherd T. M., Stanisz G. J. & Blackband S. J. Effects of temperature and aldehyde fixation on tissue water diffusion properties, studied in an erythrocyte ghost tissue model. Magnetic resonance in medicine: official journal of the Society of Magnetic Resonance in Medicine/Society of Magnetic Resonance in Medicine 56, 282–289, 10.1002/mrm.20962 (2006). 16841346

[b21] Bernus O. . Comparison of diffusion tensor imaging by cardiovascular magnetic resonance and gadolinium enhanced 3D image intensity approaches to investigation of structural anisotropy in explanted rat hearts. Journal of cardiovascular magnetic resonance: official journal of the Society for Cardiovascular Magnetic Resonance 17, 31, 10.1186/s12968-015-0129-x (2015). 25926126 PMC4414435

[b22] Plank G. . Generation of histo-anatomically representative models of the individual heart: tools and application. *Philosophical transactions*. Series A, Mathematical, physical, and engineering sciences 367, 2257–2292, 10.1098/rsta.2009.0056 (2009). PMC2881535 19414455

[b23] Cerqueira M. D. . Standardized myocardial segmentation and nomenclature for tomographic imaging of the heart. A statement for healthcare professionals from the Cardiac Imaging Committee of the Council on Clinical Cardiology of the American Heart Association. Circulation 105, 539–542 (2002). 11815441 10.1161/hc0402.102975

[b24] Sun H. Y., Tang W. N. & Wang W. M. Improving signal-to-noise ratio in magnetic resonance imaging using dynamic receiver gain. Chinese J Magn Reson 31, 515–522 (2014).

[b25] Nagy Z., Weiskopf N., Alexander D. C. & Deichmann R. A method for improving the performance of gradient systems for diffusion-weighted MRI. Magnetic resonance in medicine: official journal of the Society of Magnetic Resonance in Medicine/Society of Magnetic Resonance in Medicine 58, 763–768, 10.1002/Mrm.21379 (2007). PMC2683063 17899604

[b26] Teh I., Lohezic M., Aksentijevic D. & Schneider J. E. In Proceedings of 22nd ISMRM 2658 (2014).

[b27] Le Bihan D., Delannoy J. & Levin R. L. Temperature mapping with MR imaging of molecular diffusion: application to hyperthermia. Radiology 171, 853–857, 10.1148/radiology.171.3.2717764 (1989). 2717764

[b28] Busch J., Vannesjo S. J., Barmet C., Pruessmann K. P. & Kozerke S. Analysis of temperature dependence of background phase errors in phase-contrast cardiovascular magnetic resonance. Journal of cardiovascular magnetic resonance: official journal of the Society for Cardiovascular Magnetic Resonance 16, 97, 10.1186/s12968-014-0097-6 (2014). 25497004 PMC4263200

[b29] Angeli S. . A high-resolution cardiovascular magnetic resonance diffusion tensor map from *ex-vivo* C57BL/6 murine hearts. Journal of cardiovascular magnetic resonance: official journal of the Society for Cardiovascular Magnetic Resonance 16, 77, 10.1186/s12968-014-0077-x (2014). 25323636 PMC4198699

[b30] Jones D. K. The effect of gradient sampling schemes on measures derived from diffusion tensor MRI: A Monte Carlo study. Magnetic resonance in medicine: official journal of the Society of Magnetic Resonance in Medicine/Society of Magnetic Resonance in Medicine 51, 807–815, 10.1002/Mrm.20033 (2004). 15065255

[b31] Lombaert H. . Human atlas of the cardiac fiber architecture: study on a healthy population. IEEE transactions on medical imaging 31, 1436–1447, 10.1109/TMI.2012.2192743 (2012). 22481815

[b32] McClymont D., Teh I., Whittington H. J., Grau V. & Schneider J. E. Prospective acceleration of diffusion tensor imaging with compressed sensing using adaptive dictionaries. Magnetic resonance in medicine: official journal of the Society of Magnetic Resonance in Medicine/Society of Magnetic Resonance in Medicine 76, 248–258, 10.1002/mrm.25876 (2016). PMC4869836 26302363

[b33] Frindel C., Robini M., Schaerer J., Croisille P. & Zhu Y. M. Cardiac Fibre Trace Clustering for the Interpretation of the Human Heart Architecture. Proceedings of 5th FIMH Meeting 5528, 39–48 (2009).

[b34] Glukhov A. V. . Transmural dispersion of repolarization in failing and nonfailing human ventricle. Circulation research 106, 981–991, 10.1161/CIRCRESAHA.109.204891 (2010). 20093630 PMC2842469

[b35] Pashakhanloo F. . Myofiber Architecture of the Human Atria as Revealed by Submillimeter Diffusion Tensor Imaging. Circulation. Arrhythmia and electrophysiology 9, 10.1161/CIRCEP.116.004133 (2016). PMC7035884 27071829

[b36] Caruyer E., Lenglet C., Sapiro G. & Deriche R. Design of multishell sampling schemes with uniform coverage in diffusion MRI. Magnetic resonance in medicine: official journal of the Society of Magnetic Resonance in Medicine/Society of Magnetic Resonance in Medicine 69, 1534–1540, 10.1002/mrm.24736 (2013). PMC5381389 23625329

[b37] Schneider J. E. . High-resolution, high-throughput magnetic paragraph sign resonance imaging of mouse embryonic paragraph sign anatomy using a fast gradient-echo sequence. Magma 16, 43–51, 10.1007/s10334-003-0002-z (2003). 12695885

[b38] Tofts P. S. . Test liquids for quantitative MRI measurements of self-diffusion coefficient *in vivo*. Magnetic resonance in medicine: official journal of the Society of Magnetic Resonance in Medicine/Society of Magnetic Resonance in Medicine 43, 368–374, 10.1002/(Sici)1522-2594(200003)43:3<368::Aid-Mrm8>3.0.Co;2-B(2000 ). 10725879

[b39] Neeman M., Freyer J. P. & Sillerud L. O. A simple method for obtaining cross-term-free images for diffusion anisotropy studies in NMR microimaging. Magnetic resonance in medicine: official journal of the Society of Magnetic Resonance in Medicine/Society of Magnetic Resonance in Medicine 21, 138–143 (1991). 10.1002/mrm.1910210117 1943671

[b40] Lundell H., Alexander D. C. & Dyrby T. B. High angular resolution diffusion imaging with stimulated echoes: compensation and correction in experiment design and analysis. NMR in biomedicine 27, 918–925, 10.1002/nbm.3137 (2014). 24890716 PMC4312915

[b41] Vesanen P. T. . Temperature dependence of relaxation times and temperature mapping in ultra-low-field MRI. Journal of magnetic resonance 235, 50–57, 10.1016/j.jmr.2013.07.009 (2013). 23941818

[b42] NEMA. Determination of Signal-to-Noise Ratio (SNR) in Diagnostic Magnetic Resonance Imaging. *NEMA Standards Publication MS 1–2001* (2001).

[b43] Whitcher B., Tuch D. S., Wisco J. J., Sorensen A. G. & Wang L. Using the wild bootstrap to quantify uncertainty in diffusion tensor imaging. Human brain mapping 29, 346–362, 10.1002/hbm.20395 (2008). 17455199 PMC6870960

[b44] Wang R., Benner T., Soresen A. G. & Wedeen V. J. In Proceedings of 15th ISMRM Meeting 3720 (2007).

[b45] Basser P. J., Pajevic S., Pierpaoli C., Duda J. & Aldroubi A. *In vivo* fiber tractography using DT-MRI data. Magnetic resonance in medicine: official journal of the Society of Magnetic Resonance in Medicine/Society of Magnetic Resonance in Medicine 44, 625–632 (2000). 10.1002/1522-2594(200010)44:4<625::aid-mrm17>3.0.co;2-o 11025519

[b46] Cheng Y. Z. Mean Shift, Mode Seeking, and Clustering. Ieee T Pattern Anal 17, 790–799 (1995).

[b47] Ennis D. B., Kindlman G., Rodriguez I., Helm P. A. & McVeigh E. R. Visualization of tensor fields using superquadric glyphs. Magnet Reson Med 53, 169–176, 10.1002/mrm.20318 (2005). PMC2169197 15690516

[b48] Arts T., Costa K. D., Covell J. W. & McCulloch A. D. Relating myocardial laminar architecture to shear strain and muscle fiber orientation. American journal of physiology. Heart and circulatory physiology 280, H2222–2229 (2001). 11299225 10.1152/ajpheart.2001.280.5.H2222

[b49] Scollan D. F., Holmes A., Zhang J. & Winslow R. L. Reconstruction of cardiac ventricular geometry and fiber orientation using magnetic resonance imaging. Ann Biomed Eng 28, 934–944, 10.1114/1.1312188 (2000). 11144678 PMC1473035

[b50] Jones S. E., Buchbinder B. R. & Aharon I. Three-dimensional mapping of cortical thickness using Laplace’s equation. Human brain mapping 11, 12–32 (2000). 10997850 10.1002/1097-0193(200009)11:1<12::AID-HBM20>3.0.CO;2-K PMC6872107

