# Supplementary material for: Resolving Fine Cardiac Structures in Rats with High-Resolution Diffusion Tensor Imaging
Source: Sci Rep. 2016 Jul 28;6:30573. doi: 10.1038/srep30573 (PMC4964346; doi:10.1038/srep30573)
Supplement: Supplementary Information [file srep30573-s1.pdf]

Resolving Fine Cardiac Structures in Rats with High-Resolution Diffusion Tensor Imaging

Irvin Teh[1], Darryl McClymont[1], Rebecca A.B. Burton[2], Mahon L. Maguire[1], Hannah J. Whittington[1], Craig A. Lygate[1], Peter Kohl[3,4], and Jürgen E. Schneider[1*]

[1]Division of Cardiovascular Medicine, Radcliffe Department of Medicine, University of Oxford, Oxford, OX3 7BN, United Kingdom; [2]Department of Physiology, Anatomy and Genetics, University of Oxford, Oxford, OX1 3PT, United Kingdom; [3]National Heart and Lung Institute, Imperial College London, London, SW3 6NP, United Kingdom, [4]Institute for Experimental Cardiovascular Medicine, University Heart Centre Freiburg · Bad Krozingen, Medical School of the University of Freiburg, Freiburg, 79110, Germany.

*Email: jurgen.schneider@cardiov.ox.ac.uk

Supplementary Video 1. Eigenvector tracking in the whole heart based on $\mathbf{v_1}$. The heart is rotated 360° about its global long-axis. Tracks are colour-coded by orientation: apico-basal (red), anterior-posterior (green) and lateral-septal (blue).

Supplementary Video 2. Tracking of $\mathbf{v_3}$ in a series of axial slices from base to apex. $\mathbf{v_3}$ tracks indicate the sheet-normal direction of the myocardial laminae. In the LV, the radial arrangement of these tracks suggests that the myocardial sheets are aligned near-parallel to the myocardial wall surface in the relaxed heart. A radial arrangement of $\mathbf{v_3}$ tracks is also observed in the aorta, whereas in the RV, they are oriented more longitudinally. In the left and right atria, tracks are generally arranged normal to the local atrial surface.

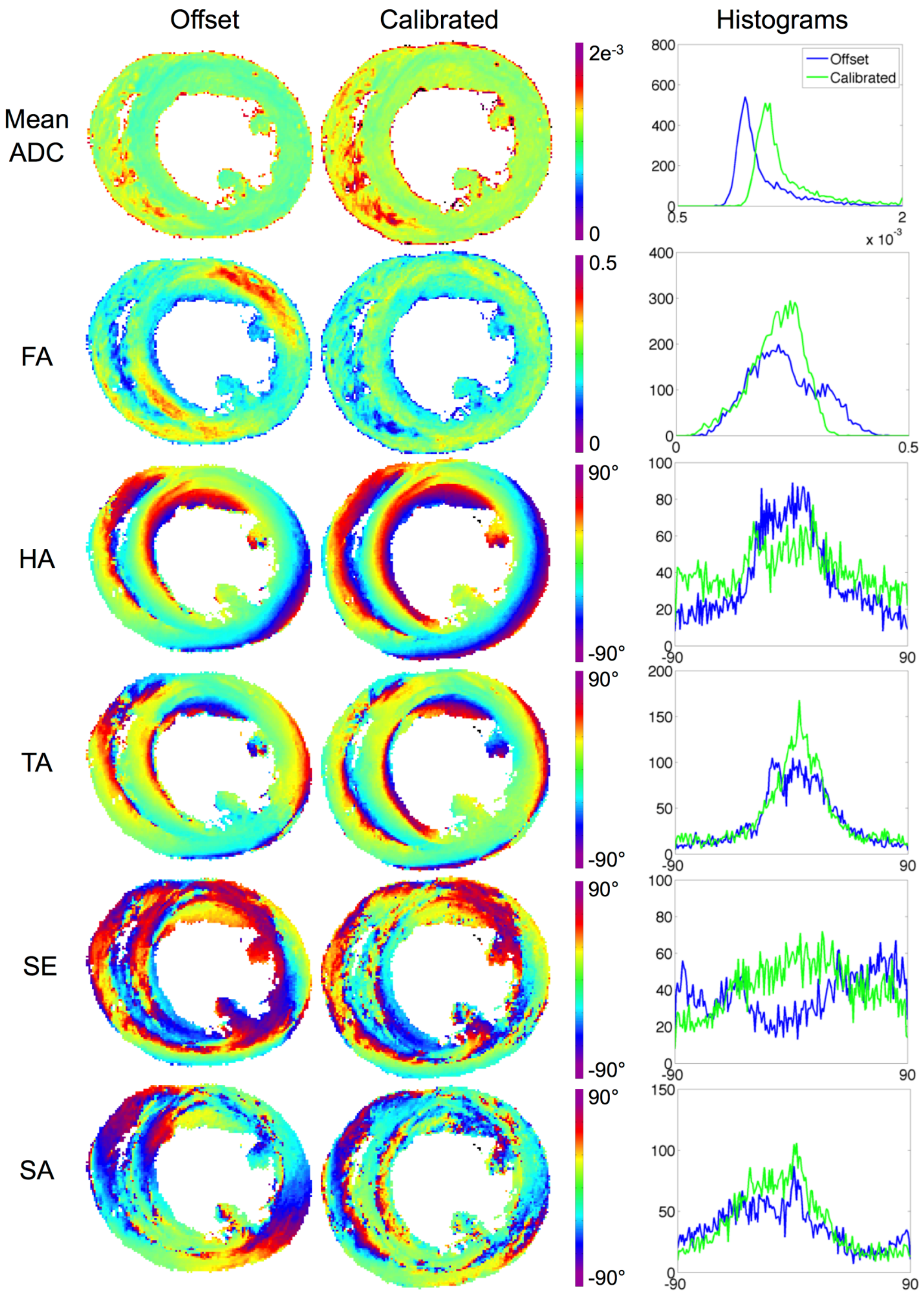

Supplementary Figure S1. Effect of gradient calibration on diffusion metrics. Maps and histograms of mean apparent diffusion coefficient (ADC), fractional anisotropy (FA), helix angle (HA), transverse angle (TA), sheet elevation (SE) and sheet azimuth (SA) in the offset and calibrated scans in a mid-ventricular short-axis slice. The 10% reduction in gradient scaling in the y (anterior-posterior) and z (apico-basal) directions resulted in corresponding compression of the heart geometry, and marked regional variations in diffusion measurements.

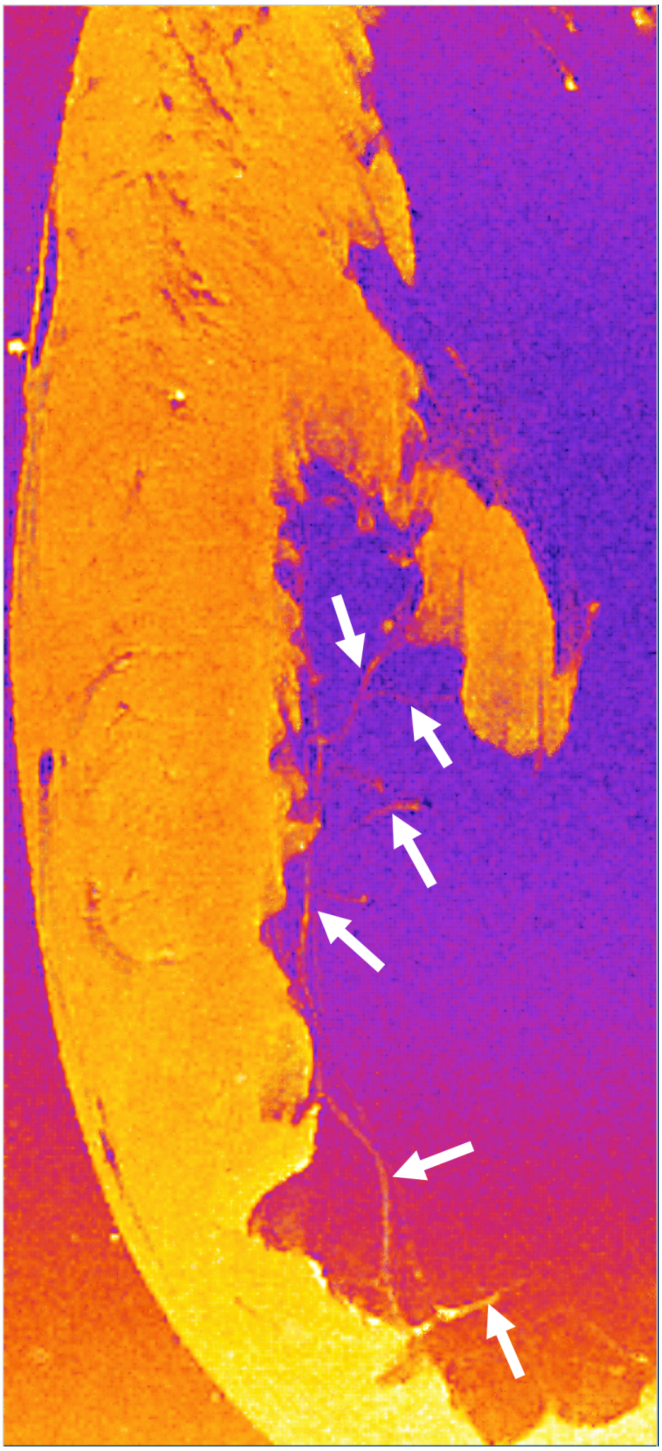

Supplementary Figure S2. Volume rendering of anatomical MRI data acquired at 33 µm isotropic resolution. A section in the posterior wall in one heart highlights putative Purkinje fibres that appear as a continuous 3D mesh-like network at the endocardial surface.
